# Leveraging nature to advance data storage: DNA as a storage medium

**DOI:** 10.1111/1751-7915.14291

**Published:** 2023-06-10

**Authors:** Kaleb Z. Abram, Zulema Udaondo

**Affiliations:** ^1^ Department of Biomedical Informatics University of Arkansas for Medical Sciences Little Rock Arkansas USA

## Abstract

Schematic overview of DNA data storage.

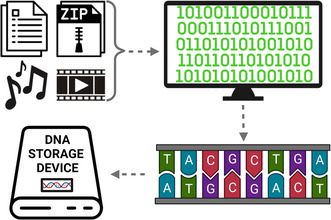

We live in a digital age which has led to ever increasing amounts of digital data about the world we live in and interact with. Today many fields are leveraging big data and machine learning to make novel discoveries and breakthroughs that would not have been possible without big data (Abram et al., 2022; Hallsworth et al., 2023; Udaondo, 2022). In parallel with the increase in research productivity enabled by big data, an additional problem is also created; namely the need for storage devices with increased storage capability (focusing on both high storage density and the longer storage device lifespans; Church et al., 2012).

One unique solution to this problem is the use of DNA as a storage medium. While DNA may initially seem like an odd choice for storing digital data, DNA has both an extremely high theoretical data density as well as an unrivaled stability when compared to more traditional data storage media. In addition, due to DNA is essential for all living organisms, enzymes that can read and write DNA are widely accessible and the DNA can remain readable as an enduring standard (Church et al., 2012). DNA has a Shannon information capacity (Erlich & Zielinski, 2017; Shannon, 2001) of 1.83 bits/nucleotide meaning that a single gram of synthetic DNA could hold hundreds of petabytes of encoded digital data (Church et al., 2012) even after considering the biochemical constraints of the use of the molecule (such as presence of high GC‐content regions and homopolymers that increases the difficulty of synthesizing and sequencing the DNA), the necessity of using barcodes, sequence dropout rate and possible degradation of DNA molecules over time (Chen et al., 2020; Erlich & Zielinski, 2017). In addition, it has been estimated that 80% of all generated data is considered *cold* (infrequently accessed i.e. digital archiving) making DNA a very promising medium for long‐term data storage (El‐Shaikh & Seeger, 2023).

In general, the process of encoding any digital data to DNA typically requires converting the digital data from binary form (base 2) into nucleotides using lookup tables (Church et al., 2012; Erlich & Zielinski, 2017; Goldman et al., 2013). These tables are designed to avoid homopolymers and long repeats which increase the difficulty to read and write DNA sequences as well as the error rate in both the reading and writing process (Church et al., 2012; Erlich & Zielinski, 2017; Goldman et al., 2013). Once the binary data is encoded into the DNA, recovering the binary data is accomplished by sequencing the DNA and using the lookup tables to convert the sequence back into binary form.

While storing information using DNA is not necessarily a new idea, it has remained largely theoretical since it was first mentioned by Richard P. Feynman in a lecture given at the annual meeting of the American Physical Society in 1959 at the California Institute of Technology (Caltech) (Feynman, 1960). Despite Feynman's lecture being purely hypothetical, it represented an important starting point for DNA as a storage medium. It took several decades after Feynman's initial proposal for the first practical efforts to utilize DNA as a storage medium which occurred in 1988 (Davis, 1996). In this work, titled Microvenus, is described how a small piece of synthetic DNA was incorporated into *Escherichia coli* using a plasmid which contained an encoded graphical icon. The DNA sequence was created by utilizing an intermediary language to convert the binary data to nucleic acids. This resulted in a double‐stranded DNA sequence containing 28 base‐pairs where 18 encoded the binary data and remaining 10 base‐pairs provided the information needed to decode the DNA sequence. While the amount of data encoded in this work was minuscule, it laid the groundwork for subsequent efforts in this area.

After Microvenus, multiple efforts (Bancroft et al., 2001; Clelland et al., 1999; Gustafsson, 2009; Portney et al., 2008; Wong et al., 2003; Yachie et al., 2007) to increase the potential of DNA as a storage medium were undertaken with a range of encoded bits from 12 to 1688. In 2010, the first synthetic genome of *Mycoplasma mycoides* JCVI‐syn1.0, was created with ‘watermark’ sequences which encoded 7920 bits of data into DNA (Church et al., 2012; Gibson et al., 2010), the largest amount of data encoded in DNA at that time. Generally speaking, these efforts were limited by the difficulty in writing and reading long DNA sequences without errors as well as the overall cost for both synthesizing and sequencing the DNA.

Over the past decade, advances in molecular biology have led to an exponential increase in the potential use of DNA as a storage medium. In 2012, George Church, Yuan Gao, and Sriram Kosuri published the first ‘real’ application of DNA as a data storage medium (Church et al., 2012). In this work, Church and colleagues were able to encode the following digital data onto a high‐fidelity DNA microchip: a 53,426 word book (659 kb), 11 JPG images, and a JavaScript program. Both the density and the methodology employed by Church had several advantages over the methodologies employed by previous works. One of the most important changes was the use of a single bit encoded per nucleotide, where adenine and cytosine encoded binary zero, and guanine and thymine encoded binary one. The importance of this change comes from the ability to encode the same data in several different combinations which enables the avoidance of sequences that are difficult to synthesize or sequence (such as homopolymers or high GC‐content regions). Another important change was the use of next‐generation technologies for both the DNA synthesis and sequencing which resulted in approximately 100,000‐fold decrease in cost compared to previous encoding efforts (Church et al., 2012).

The next major milestone in the use of DNA as a storage medium occurred in 2017 with DNA Fountain (Erlich & Zielinski, 2017). In this study, the authors devised a strategy for the use of DNA as a storage medium in which they achieved the highest realized capacity of DNA storage to date (1.57 bits/nucleotide). The realized capacity is the ratio between the amount of information that can be stored and the theoretical Shannon information capacity of the data medium. This increase in realized capacity represented a 24% increase in realized capacity compared to the next best methodology at this time. Moreover, due to the methodology employed by the authors, the perfect retrieval of the digital data was possible from an equivalent sequence coverage of a single tile of Illumina sequencing. The authors also tested a data retrieval process that could be performed an almost unlimited number of times from the original DNA sample.

One of the limitations to DNA data storage at this point in time, was the lack of random access which is a hallmark of current storage media for digital data. In order to read a single file encoded on a given DNA data storage medium, all files encoded on the DNA would need to be sequenced and decoded to access that file. In 2018, the first solution to this limitation was published by Organick et al. (2018) in which the authors encoded over 200 MB of unique (compressed) data in 35 distinct files and perfectly retrieved them via a random access approach. To achieve random access in large DNA data storage systems, the design of PCR primers that reliably amplify a given file without crosstalk was of paramount importance (Organick et al., 2018). The methodology utilized in this work was able to produce primers which were designed to have a minimum of 30% unique sequence composition compared to other primers, avoiding secondary structure formation and long homopolymers, and having a narrow range of melting temperatures (55–60°C). Another major advancement of this work over previous works was the coding algorithm used which was explicitly designed to reduce sequencing redundancy, reducing both the number of physical copies of oligonucleotides and the amount of sequencing resources needed to fully recover the encoded data.

Recent advances in DNA data storage have brought this technology closer to practical use. However, one of the main limitations of using DNA as a digital data storage medium is the need for highly skilled specialists both to incorporate the data and extract and interpret it. Thus, compared to traditional storage media, the amount of expertise required to utilize DNA as a storage medium could make DNA a non‐viable option for broad utilization. To address this issue, Takahashi and colleagues presented in 2018 (Takahashi et al., 2019), the first fully automated end‐to‐end DNA data storage device. While the device serves as a proof of concept, it represents an important step forward towards the use of DNA as a standard storage medium. This system has modular design that also enables the improvement of the individual components of the automated device without explicitly needing to improve the whole system. The DNA storage device presented in the work by Takahashi et al., is comprised of three major components to accomplish both the read and write process: an encode/decode software module, a DNA synthesis module, and a DNA preparation and sequencing module. The entire device can fit on a benchtop and costs approximately $10,000 USD, though this cost could in theory be lowered to around $4000 USD using precise calibration and elimination of costly sensors and actuators.

Other complications of DNA as a storage medium is the reliance on chemically synthesizing DNA which is both time consuming and produces toxic byproducts (Lee et al., 2020; LeProust et al., 2010). In 2020, two different non‐chemical DNA synthesis approaches were proposed to create DNA for data storage: multiplexed enzymatic DNA synthesis leveraging directed photons (Lee et al., 2020) and enzymatic nicking of native DNA sequences (Tabatabaei et al., 2020). Although up until this point, the enzymatic synthesis of DNA had not been demonstrated in a parallelized fashion, the work by Lee et al. presents a multiplexed enzymatic DNA synthesis methodology which leverages photolithography to synthesize DNA in a spatially‐selective fashion on an array surface. However, this methodology does not address some of the shortcomings of DNA as a storage medium, such as high cost or read‐write latency, when compared to traditional storage devices due to the need to synthesize DNA. The work by Tabatabaei et al., presents a synthesis free methodology which leverages readily available native DNA and enzymatic nicking to encode data. The novelty in the methodology lies in the use of enzymes to nick the DNA at predetermined positions along the backbone of native double‐stranded DNA creating a ‘DNA punch card’ with the encoded data. Moreover, due to the presence/absence pattern of the nicks used to encode the data, this methodology is more similar to the native binary encoding of digital data where 0 and 1 are used to encode information. To decode the information, the data can be decoded from the nicked DNA using next‐generation sequencing techniques, as is done in synthesis‐based DNA storage methods, followed by the read alignment of the sequenced DNA against the reference (non‐nicked DNA) using read coverage to determine the nicked positions.

While these publications are vital to the development of DNA storage methods, most DNA storage methods do not yet provide a systematic approach for writing and reading random data objects. Recent efforts have been undertaken to address these limitations such as the novel object‐based storage architecture DNAContainer. The work by Alex El‐Shaikh and Bernhard Seeger, published in 2023 (El‐Shaikh & Seeger, 2023), presents a DNA storage architecture via microarrays that provides a large enough virtual address space to enable random access at a large scale while still staying within the biochemical constraints of DNA. Moreover, this work also enables the implementation of common external data structures (i.e. arrays and lists) to store data in fixed size blocks.

As depicted by the works highlighted above, despite the major differences between traditional storage media and DNA, there has been an increasing rate of development in this area during the past decade. Among the strengths of the use of DNA data storage are, the durability and the stability of the storage media (DNA), the high information density, and the low maintenance cost of the storage medium. Moreover, recent advances in both sequencing accuracy and decreased cost have also increased the feasibility of DNA data storage without directly relying on optimization of existing DNA data storage methodologies. Although the use of DNA as a storage medium currently has some fairly significant limitations compared to traditional storage solutions, exciting and rapid advances in this field may lead to solutions or mitigations of these existing limitations in the near future, that could take us into a new era of carbon‐based storage systems.

## AUTHOR CONTRIBUTIONS

**Kaleb Z. Abram:** Conceptualization (equal); investigation (equal); methodology (equal); supervision (equal); writing – original draft (equal); writing – review and editing (equal). **Zulema Udaondo:** Conceptualization (equal); investigation (equal); methodology (equal); supervision (equal); writing – original draft (equal); writing – review and editing (equal).

## CONFLICT OF INTEREST STATEMENT

None declared.
